# Drug-coated balloon-based versus drug-eluting stent-only revascularization in patients with diabetes and multivessel coronary artery disease

**DOI:** 10.1186/s12933-023-01853-0

**Published:** 2023-05-20

**Authors:** Ae-Young Her, Eun-Seok Shin, Sunwon Kim, Bitna Kim, Tae-Hyun Kim, Chang-Bae Sohn, Byung Joo Choi, Yongwhi Park, Jung Rae Cho, Young-Hoon Jeong

**Affiliations:** 1 Division of Cardiology, Department of Internal Medicine, Kangwon National University College of Medicine, Kangwon National University School of Medicine, Chuncheon, South Korea; 2 Department of Cardiology, Ulsan University Hospital, University of Ulsan College of Medicine, 877 Bangeojinsunhwan-doro, Dong-gu, Ulsan, 44033 South Korea; 3 Department of Cardiology, Korea University Ansan Hospital, Ansan-si, South Korea; 4 Department of Cardiology, Ulsan Medical Center, Ulsan, South Korea; 5 Department of Internal Medicine, Cardiovascular Center, Gyeongsang National University School of Medicine, Gyeongsang, South Korea; 6 Cardiology Division, Department of Internal Medicine, Kangnam Sacred Heart Hospital, Hallym University College of Medicine, Seoul, South Korea; 7 Division of Cardiology, Chung-Ang University Gwangmyeong Hospital, Chung-Ang University College of Medicine, Gwangmyeong, South Korea

**Keywords:** Diabetes mellitus, Multivessel, Drug-coated balloon, Drug-eluting stent, Coronary artery disease, Percutaneous coronary intervention

## Abstract

**Background:**

Data on drug-coated balloon (DCB) treatment in the context of diabetes mellitus (DM) and multivessel coronary artery disease (CAD) are limited. We aimed to investigate the clinical impact of DCB-based revascularization on percutaneous coronary intervention (PCI) in patients with DM and multivessel CAD.

**Methods:**

A total of 254 patients with multivessel disease (104 patients with DM) successfully treated with DCB alone or combined with drug-eluting stent (DES) were retrospectively enrolled (DCB-based group) and compared with 254 propensity-matched patients treated with second-generation DES from the PTRG-DES registry (n = 13,160 patients) (DES-only group). Major adverse cardiovascular events (MACE) comprised cardiac death, myocardial infarction, stroke, stent or target lesion thrombosis, target vessel revascularization, and major bleeding at 2 years.

**Results:**

The DCB-based group was associated with a reduced risk of MACE in patients with DM (hazard ratio [HR] 0.19, 95% confidence interval [CI] 0.05–0.68, p = 0.003], but not in those without DM (HR 0.52, 95% CI 0.20–1.38, p = 0.167) at the 2-year follow-up. In patients with DM, the risk of cardiac death was lower in the DCB-based group than the DES-only group, but not in those without DM. In both patients with or without DM, the burdens of DES and small DES (less than 2.5 mm) used were lower in the DCB-based group than in the DES-only group.

**Conclusions:**

In multivessel CAD, the clinical benefit of a DCB-based revascularization strategy appears to be more evident in patients with DM than in those without DM after 2 years of follow-up. (Impact of Drug-Coated Balloon Treatment in De Novo Coronary Lesion; NCT04619277)

## Introduction

Patients with diabetes mellitus (DM) undergoing percutaneous coronary intervention (PCI) have worse clinical outcomes, such as increased risk of in-stent restenosis (ISR), stent thrombosis, myocardial infarction, and death, compared with that of patients without DM [1–3]. Furthermore, patients with DM often have disease that is diffuse, long, and multivessel, and they require multivessel revascularization by either PCI or coronary artery bypass graft (CABG) [4, 5]. Although PCI with drug-eluting stent (DES) has significantly reduced the rates of repeat revascularization in patients with coronary artery disease (CAD), PCI with DES for multivessel disease in patients with DM has been challenging as a revascularization option.

Drug-coated balloon (DCB) treatment leaves nothing of lesions behind, and it reduces the risk of stent-associated maladaptive biologic responses causative of restenosis and thrombosis, and allows for favorable natural vascular healing [6, 7]. In particular, using DCB or combined with DES as part of a hybrid procedure (DCB-based revascularization strategy) to reduce stent burden (stent length or number) may be an alternative and useful treatment approach for multivessel disease. Recently, we reported the benefits of a DCB-based revascularization strategy for multivessel PCI involving DCB used alone or in combination with DES that resulted in a reduced stent burden compared to a DES-only treatment group [8]. However, the benefit of DCB-based revascularization for multivessel CAD in the patients with DM has not been fully verified in the contemporary DES era. Therefore, we sought to evaluate the clinical impact of a DCB-based revascularization strategy in patients with DM and multivessel disease who underwent PCI with contemporary DES.

## Methods

### Patient population

A total of 254 patients with successful PCI for multivessel CAD including patients with DM who received DCB alone or in combination with DES were retrospectively enrolled between 2012 and 2020 from three teaching hospitals in South Korea (Ulsan University Hospital, Ulsan Medical Center, and Korea University Ansan Hospital) with experienced physicians providing treatment for patients with multivessel CAD using DCB (Impact of Drug-coated Balloon Treatment in de Novo Coronary Lesion; NCT04619277). Eligible patients were those who had lesions with ≥ 50% narrowing and who the investigator considered to require PCI for two or more major epicardial coronary lesions. Patients with DM were defined as patients with a history of DM under medication or fasting plasma glucose ≥ 126 mg/dL. All patients were diagnosed with Type 2 DM in this study. Patients were excluded from the analysis if they had previously undergone CABG; presented with cardiogenic shock, thrombolysis before PCI, single-vessel disease, or suboptimal or failed PCI for target lesions; or were lost to follow-up. Additionally, patients’ vessels were required to be sufficiently large to accommodate DES implantation. The results of the hybrid approach in these patients were compared with those of 254 propensity-matched patients from the PTRG-DES consortium, who were treated with DES-only (https://www.clinicaltrials.gov) (unique identifier: NCT04734028). This consortium combined nine prospective registries from 32 Korean academic centers, contributing data from 13,160 patients who were treated with DES between July 2003 and August 2018 [9]. Out of a total of 13,160 PTRG-DES consortium patients, 11,226 patients received second-generation DES, and among them, 4,460 patients underwent multivessel DES implantation. Propensity score matching was performed for 4,427 patients, excluding 33 patients who had previously undergone CABG.

The study protocol was approved by the institutional review board of each participating center, and all patients provided written informed consent at the time of enrollment.

### Procedure

For patients with multivessel disease, the PCI target lesions were first determined, then balloon angioplasty was performed to determine whether DCB treatment would be possible. The DCB-based treatment group received interventions performed according to international and Asia-Pacific consensus recommendations for DCB treatment [10, 11]. Specifically, predilation with a plain balloon at the recommended balloon-to-vessel ratio of 0.8 to 1.0 was mandatory. After predilation balloon angioplasty, stenting was deferred for all types of dissections (A to E), provided that thrombolysis in myocardial infarction (TIMI) grade 3 flow had been achieved. In cases of flow-limiting dissection after predilation (TIMI flow grade < 3) and > 30% visual residual stenosis, PCI with stent implantation without use of a DCB was recommended. As an exception, even with normal (i.e., TIMI grade 3) flow and residual diameter stenosis ≤ 30%, the operator could choose to use either DES or DCB if the patient complained of new-onset chest pain after balloon angioplasty or if a change in the ST-segment or progression of dissection was noted [8]. The DCB was inflated to its nominal pressure for at least 60 s, taking care to extend it at least 2 mm beyond the predilation balloon length. All DCB were coated with 3.0 µg/mm^2^ paclitaxel combined with iopromide (SeQuent Please© by B. Braun, Germany), as a carrier for the drug. After DCB use, the final assessment was performed at least 5 min after administering a bolus of an intracoronary vasodilator, to prevent any remaining acute vessel closure. In cases of high thrombus burden, a bailout glycoprotein IIb/IIIa receptor inhibitor strategy was used. The duration of the prescribed dual antiplatelet therapy was at the discretion of the attending physician.

### Clinical follow-up and endpoints

All 508 patients underwent a clinical follow-up following the index procedure via telephone interviews and outpatient clinic visits. The study endpoint was cumulative major adverse cardiac events (MACE) at 2 years, a composite of cardiac death, myocardial infarction (MI), stroke, probable or definite stent or target lesion thrombosis, target vessel revascularization (TVR), and major bleeding. Cardiac death was defined as any death that was not clearly of extracardiac origin, including MI, according to previously published guidelines [12]. Additionally, probable or definite stent or target lesion thrombosis was defined according to the definition by the Academic Research Consortium [13], and major bleeding was defined as Bleeding Academic Research Consortium type 3 to 5 bleeding [14].

### Statistical analysis

Clinical characteristics are reported as percentages for categorical variables and as means with standard deviations for continuous variables. Comparisons between groups were made using either Pearson’s chi-squared test or Fisher’s exact test for categorical variables, and Student’s t-test for continuous variables, as appropriate. In comparing clinical outcomes between the groups, the cumulative incidences of MACE and other outcomes were estimated using the Kaplan–Meier method, and the curves were compared using the log-rank test. To reduce the effect of potential confounding factors, we used propensity score matching to adjust for differences in baseline characteristics. The propensity score was estimated using logistic regression by considering demographic and clinical variables (age, sex, hypertension, DM, current smoking, end-stage renal disease, previous history of MI, previous history of PCI, left main disease, presentation of acute MI, chronic total occlusion, total number of treated vessels, total number of devices used, total length of devices used, and mean diameter of devices used). Without setting the caliper size (R default caliper size = NULL), patients were 1:1 matched using the nearest-neighbor method with respect to the calculated score. All p-values were two-sided, and a value of < 0.05 was considered statistically significant. R version 4.1.2 (R Foundation for Statistical Computing, Vienna, Austria) was used for all statistical analyses in this study.

## Results

**Fig. 1:**
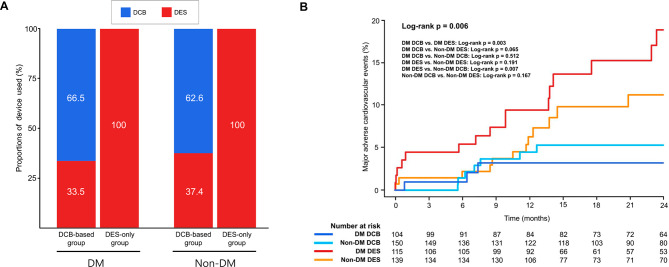
Clinical impact of DCB-based PCI in patients with DM and multivessel CAD **A.** Proportions of DCB and DES devices used in the DM and non-DM groups **B.** Cumulative incidence of MACE during 2 years of follow-up by treatment strategy and the presence of DM

Among a total of 508 patients with multivessel disease, 219 patients (43.1%) composed the DM group, and 289 patients (56.9%) composed the non-DM group. DCB-based treatment was performed in 47.5% (n = 104) of the DM group and 51.9% (n = 150) of the non-DM group. The baseline clinical and procedural characteristics of the patients are described by DM group and treatment strategy in Table 1. In the DM group, those receiving DCB-based compared to DES-only treatment had lower total number of DES used (n = 0.9 for DCB-based vs. n = 2.5 for DES-only; p < 0.001), shorter total length of DES (21.5 mm for DCB-based vs. 64.9 mm for DES-only; p < 0.001), larger mean diameter of DES (3.2 mm for DCB-based vs. 2.8 mm for DES-only; p < 0.001), and less use of small DES (≤ 2.5 mm) (10.1% for DCB-based vs. 42.6% for DES-only; p < 0.001). In the non-DM group, those receiving DCB-based compared to DES-only treatment showed less presentation of stable angina (25.3% for DCB-based vs. 38.1% for DES-only; p = 0.027), lower total number of DES used (n = 1.0 for DCB-based vs. n = 2.6 for DES-only; p < 0.001), shorter total length of DES (24.4 mm for DCB-based vs. 63.6 mm for DES-only; p < 0.001), larger mean diameter of DES (3.3 mm for DCB-based vs. 2.8 mm for DES-only; p < 0.001), and less use of small DES (≤ 2.5 mm) (6.1% for DCB-based vs. 43.2% for DES-only; p < 0.001). Of patients in the DM group receiving DCB-based treatment, 33.7% were treated with DCB alone and 66.3% were treated with the hybrid approach combining DCB and DES, while of those in the non-DM group who received DCB-based treatment, 34.7% were treated with DCB alone and 65.3% were treated with the hybrid approach. For those receiving DCB-based treatment, the number of stents used was significantly reduced (by 66.5% and 62.6% in the DM and non-DM groups, respectively [Fig. 1A]) compared to those receiving the DES-only treatment.

**Table 1:** Clinical and procedural characteristics of the patients according to DM and treatment strategy

	DM (n = 219)				Non-DM (n = 289)		
	DCB-based treatment	DES-only treatment	p Value		DCB-based treatment	DES-only treatment	p Value
	(n = 104)	(n = 115)			(n = 150)	(n = 139)	
Age, years	64.3 ±9.2	64.4 ±10.8	0.926		62.1 ±10.6	63.7 ±11.1	0.219
Men	75 (72.1)	73 (63.5)	0.223		111 (74.0)	96 (69.1)	0.424
Hypertension	84 (80.8)	96 (83.5)	0.729		97 (64.7)	94 (67.6)	0.684
Smoking	36 (34.6)	33 (28.7)	0.426		51 (34.0)	49 (35.3)	0.921
Prior MI	8 (7.7)	12 (10.4)	0.639		17 (11.3)	19 (13.7)	0.673
Prior PCI	13 (12.5)	21 (18.3)	0.323		25 (16.7)	19 (13.7)	0.586
End-stage renal disease	9 (8.7)	12 (10.4)	0.828		3 (2.0)	4 (2.9)	0.919
Clinical presentation							
Stable angina	34 (32.7)	44 (38.3)	0.473		38 (25.3)	53 (38.1)	**0.027**
Unstable angina	47 (45.2)	37 (32.2)	0.066		64 (42.7)	45 (32.4)	0.093
Acute myocardial infarction	23 (22.1)	34 (29.6)	0.271		48 (32.0)	41 (29.5)	0.739
DCB-only treatment	35 (33.7)	0	-		52 (34.7)	0	-
Target lesion and procedure characteristics							
Left main	11 (10.6)	17 (14.8)	0.467		21 (14.0)	23 (16.5)	0.661
LAD	83 (79.8)	89 (77.4)	0.787		111 (74.0)	109 (78.4)	0.458
LCX	80 (76.9)	69 (60.0)	**0.011**		119 (79.3)	94 (67.6)	**0.034**
RCA	54 (51.9)	80 (69.6)	**0.011**		82 (54.7)	83 (59.7)	0.455
Chronic total occlusion	20 (19.2)	26 (22.6)	0.655		32 (21.3)	22 (15.8)	0.294
Total number of diseased vessel	2.4 ± 0.5	2.4 ± 0.5	0.447		2.4 ± 0.5	2.4 ± 0.5	0.876
Total number of treated vessel	2.2 ± 0.4	2.2 ± 0.4	0.987		2.2 ± 0.4	2.2 ± 0.4	0.356
Total number of device used	2.6 ± 0.8	2.5 ± 0.9	0.744		2.6 ± 1.0	2.6 ± 0.9	0.727
Total device length, mm	66.1 ± 23.8	64.9 ± 30.6	0.741		64.6 ± 26.6	63.6 ± 29.9	0.760
Device diameter, mm	2.8 ± 0.2	2.8 ± 0.4	0.222		2.8 ± 0.3	2.8 ± 0.4	0.613
Total number of DCB used	1.7 ± 0.8	0			1.6 ± 0.8	0	
Total DCB length, mm	44.5 ± 23.9	0			40.2 ± 23.4	0	
DCB diameter, mm	2.6 ± 0.2	0			2.6 ± 0.3	0	
Small DCB used (diameter ≦ 2.5 mm)	64/104 (61.5)	0			96/150 (64.0)	0	
Total number of DES used	0.9 ± 0.8	2.5 ± 0.9	**< 0.001**		1.0 ± 0.9	2.6 ± 0.9	**< 0.001**
Total DES length, mm	21.5 ± 20.7	64.9 ± 30.6	**< 0.001**		24.4 ± 24.7	63.6 ± 29.9	**< 0.001**
DES diameter, mm	3.2 ± 0.5	2.8 ± 0.4	**< 0.001**		3.3 ± 0.5	2.8 ± 0.4	**< 0.001**
Small DES used ( ≦ 2.5 mm)	7/69 (10.1)	49/115 (42.6)	**< 0.001**		6/98 (6.1)	60/139 (43.2)	**< 0.001**

Values are presented as the mean ± SD or n (%)

DM = diabetes mellitus; DCB = drug-coated balloon; DES = drug-eluting stent; MI = myocardial infarction; PCI = percutaneous coronary intervention; LAD = left anterior descending artery; LCX = left circumflex artery; RCA = right coronary artery

**Fig. 2:**
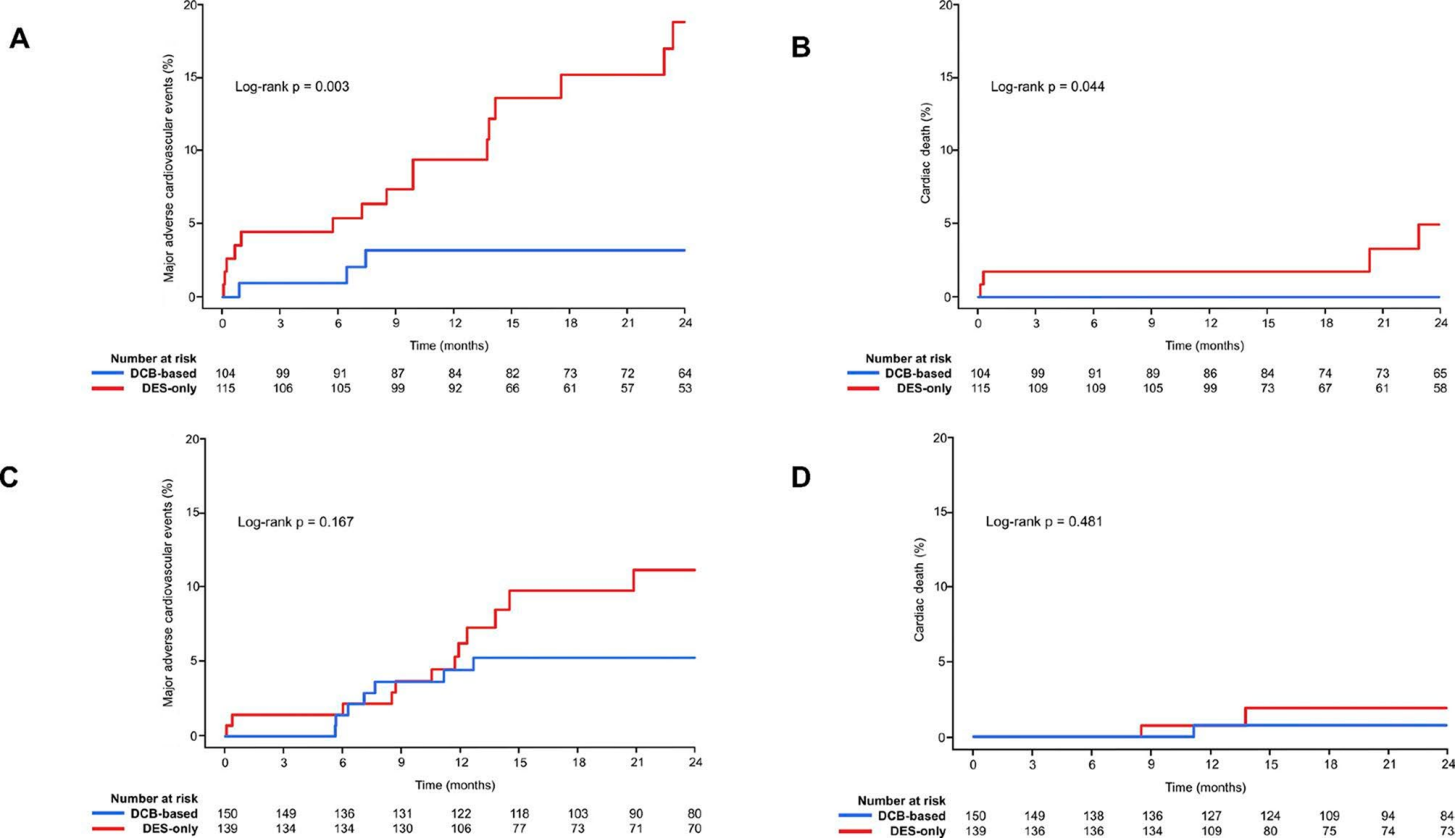
Cumulative incidence of MACE and cardiac death after DCB-based and DES-only revascularization in the DM group (**A, B**) and the non-DM group (**C, D**)

Table 2 shows the comparison of the cumulative incidences of major clinical outcomes between groups for the 2-year follow-up period (interquartile range [IQR]: 1.1–4.5 years). In the DM group, those receiving DCB-based treatment had both a significantly lower cumulative incidence of MACE at 2 years than those in the DES-only treatment (n = 3 [2.9%] for DCB-based vs. n = 16 [13.9%] for DES-only; hazard ratio [HR]: 0.19; 95% confidence interval [CI]: 0.05–0.68; log-rank p = 0.003) (Table 2; Fig. 2A, and Fig. 1B) and a significantly lower incidence of cardiac death compared to those receiving DES-only treatment (n = 0 for DCB-based vs. n = 4 [3.5%] for DES-only; log-rank p = 0.044) (Table 2; Fig. 2B). However, in the non-DM group, the cumulative incidences of MACE and cardiac death did not significantly differ for those receiving DCB-based compared to DES-only treatment (MACE: n = 7 [4.7%] for DCB-based vs. n = 12 [8.6%] for DES-only [HR: 0.52; 95% CI: 0.20–1.38; log-rank p = 0.167]; cardiac death: n = 1 [0.7%] for DCB-based vs. n = 2 [1.4%] for DES-only [HR: 0.43; 95% CI: 0.03–5.34; log-rank p = 0.481]) (Table 2; Fig. 2C and D, and Fig. 1B). There were no cases of MI or target lesion thrombosis in patients receiving DCB-based treatment in either the DM or the non-DM group.

**Table 2:** Comparison of clinical outcomes between DCB-based treatment and DES-only treatment according to the presence of DM at 2 years follow-up

	DM (n = 219)					Non-DM (n = 289)			
	DCB-based treatment	DES-only treatment	HR (95% CI)	p Value*		DCB-based treatment	DES-only treatment	HR (95% CI)	p Value*
	(n = 104)	(n = 115)				(n = 150)	(n = 139)		
MACE	3 (2.9)	16 (13.9)	0.19 (0.05–0.68)	**0.003**		7 (4.7)	12 (8.6)	0.52 (0.20–1.38)	0.167
Cardiac death	0	4 (3.5)	-	**0.044**		1 (0.7)	2 (1.4)	0.43 (0.03–5.34)	0.481
Myocardial infarction	0	1 (0.9)	-	0.290		0	2 (1.4)	-	0.155
Stroke	0	1 (0.9)	-	0.333		0	0	-	-
Stent or target lesion thrombosis	0	0	-	-		0	1 (0.7)	-	0.316
Target vessel revascularization	2 (1.9)	8 (7.0)	0.27 (0.05–1.34)	0.077		6 (4.0)	8 (5.8)	0.69 (0.23–2.07)	0.492
Major bleeding	1 (1.0)	4 (3.5)	0.26 (0.03–2.34)	0.196		0	3 (2.2)	-	0.063

Values are presented as n (%). p value* was obtained from the log-rank test

MACE was composed of cardiac death, myocardial infarction, stroke, stent or target lesion thrombosis, target vessel revascularization, and major bleeding (Bleeding Academic Research Consortium bleeding type 3 or greater)

DM = diabetes mellitus; DCB = drug-coated balloon; DES = drug-eluting stent; MACE = major adverse cardiovascular events

## Discussion

The main findings of this study were as follows: (1) in patients with DM, DCB-based treatment significantly reduced the risk of MACE and cardiac death compared with DES-only treatment for de novo multivessel CAD at 2-year follow-up; (2) in patients with non-DM, the clinical outcomes were similar with both DCB-based treatment and DES-only treatment for multivessel CAD. Therefore, a DCB-based revascularization strategy may be an acceptable approach for patients with DM and multivessel CAD.

DM accelerates atherosclerosis in multiple vascular beds and is associated with a significantly higher risk of CAD, and its prevalence is still growing globally [15]. It has been demonstrated that CAD in the DM population is more likely to involve diffuse and multivessel disease and is associated with more severe cardiovascular events and worse clinical outcomes. DM is also associated with adverse stent-related outcomes after PCI, with increased risk of stent restenosis and thrombotic obstruction [16–18]. A pooled analysis of the BIO-RESOR and BIONYX trials demonstrated that patients with DM had higher risks of target lesion failure than patients without DM after PCI [18]. Thus, PCI is expected to be more challenging and have potentially worse outcomes in the DM population. Furthermore, the higher risk of both adverse patient-related and stent-related outcomes raises concerns about whether aggressive revascularization is beneficial in the setting of DM.

The current guidelines suggest that for patients with DM and multivessel CAD, revascularization with CABG might be the preferred approach [5, 19]. In PCI with DES, the rates of new MI and repeat revascularization procedures for new lesions are significantly higher for PCI using DES than for CABG. Protection from both new MI and the need for repeat revascularization has been suggested to be the main mechanism of benefit of CABG in patients with diffuse atherosclerosis such as in DM [20, 21]. These explanations are consistent with the results of recent observational and meta-analysis studies comparing multivessel PCI with DES and CABG [22–24]. Additionally, a recent study showed that the clinical long-term benefit of complete revascularization with relief of residual CAD is more prominent in patients with versus without DM (POCO [patient-oriented composite outcome]; aHR: 0.70; 95% CI: 0.52–0.93, p = 0.016) [25]. However, stent implantation at all visible coronary lesions is not practical or appropriate. According to the results of this study, although the total number of treated vessels and the number of devices used were comparable in the DCB-based group and the DES-only group in DM patients, MACE was better in the DCB-based group. This shows that the target lesions in multivessel disease can be treated similarly to the DES-only group while reducing the stent burden, and that the outcome can be improved. When DCB-based treatment is applied to PCI, further research is needed to see how much outcome improvement can be achieved compared to CABG.

The advantages of DCB treatment include homogeneous drug delivery to the vessel wall, immediate drug release without the use of a polymer, and the freedom of leaving no foreign object behind in the vessel. The DCB treatment, involving no-metallic stent struts or polymer, may reduce intimal hyperplasia and vessel inflammation, preserving vessel anatomy and flow compared with DES. Furthermore, although the exact mechanism of late lumen increase is not well understood, DCB treatment of de novo coronary lesions after predilation was known to lead to late lumen enlargement. Therefore, considering the nature of DM in CAD, a DCB-based strategy (DCB alone or combined with DES) may be a good alternative to a DES-only strategy in treating multivessel CAD in patients with DM.

In the present study, we showed that in patient with DM who had multivessel CAD, DCB-based treatment was significantly associated with a lower risk of MACE and cardiac death than DES-only treatment; however, this association was not seen in patients without DM. Although it showed no statistical significance, we demonstrated that the need for TVR in the DM group was numerically lower with DCB-based treatment compared with DES-only treatment (1.9% vs. 7.0%; aHR: 0.23; 95% CI: 0.03–1.46; log-rank p = 0.077). Our study results are consistent with those of previous studies on the long-term clinical impact regarding TVR of DCB versus DES treatment in patients with DM having de novo coronary lesions [26]. A previous subgroup analysis of the BASKET-SMALL 2 trial with 3 years of follow-up demonstrated that in patients with DM, rates of TVR were significantly lower in the DCB compared to the DES group (9.1% for DCB vs. 15.0% for DES; HR: 0.40; 95% CI: 0.17–0.94; p = 0.036 [26]; P for interaction = 0.011), but not in patients without DM.

Patients with DM have a relatively smaller vessel caliber, with longer and more diffuse de novo lesions, compared to patients who do not have DM [27]. This makes it challenging to choose an appropriate stent size and length to cover the entire disease segment, leading to varying degrees of geographical miss at the initial PCI; it further predisposes the patients to the development of restenosis and thrombosis [28]. In this context, the clinical benefit of DCB-based treatment for patients with DM seems to be due to reduction of the risk associated with small-sized DES through treatment of small vessel lesions without stenting. Our results suggest that DCB use can be an alternative approach to DES for the treatment of DM with multivessel CAD, either alone for smaller coronary vessels, or in combination with DES for large lesions. Further studies are required for comprehensive evaluation of the role of DCB in this setting.

There are some limitations in this study. First, this study has the innate limitations of its observational nature and the use of registry data. Laboratory test results such as glycosylated hemoglobin A1c (HbA1c) during the follow-up period, which may be associated with DM management status and could impact the outcome, but we could not provide these data. In addition, leaving the choice of treatment strategy to the discretion of the physician inevitably introduces the limitation of selection bias. We addressed this issue by applying extensive sensitivity analyses in which measured or unmeasured confounders were adjusted to minimize the bias from different baseline characteristics. Second, each patient enrolled in this study was treated at an expert center in DCB-only treatment for de novo CAD. Thus, these results may not be reproducible without an adequate learning curve. Third, differences between the enrollment periods of the two groups might have led to differences in results related to technological changes. However, although the PTRG-DES registry was established in 2003, the patients whose data were used in the propensity match analysis had received second-generation DES. Therefore, differences between groups related to device development and PCI technique improvement are not expected to be significant. Further prospective randomized non-inferiority or superiority clinical trials with larger numbers of patients are needed to evaluate long-term outcomes after DCB-based treatment in patients with DM and multivessel CAD.

## Conclusion

In multivessel CAD, a DCB-based treatment approach (DCB alone or combined with DES) was associated with a reduced risk of MACE in patients with DM, but not in patients without DM. The role of DCB in this setting should be assessed in prospective randomized controlled trials.

## Data Availability

Not applicable.

## Abbreviations and Acronyms

DM: Diabetes mellitus
PCI: Percutaneous coronary intervention
ISR: In-stent restenosis
CABG: Coronary artery bypass graft
DES: Drug-eluting stent
CAD: Coronary artery disease
DCB: Drug-coated balloon
TIMI: Thrombolysis in myocardial infarction
MACE: Major adverse cardiovascular events
TVR: Target vessel revascularization
MI: Myocardial infarction
POCO: Patient-oriented composite outcome
